# Some Observations on the Use of the Tampon in the Hemorrhage of Abortion

**Published:** 1882-12

**Authors:** Rufus W. Griswold

**Affiliations:** Rocky Hill, Conn.


ARTICLE II.

### SOME OBSERVATIONS ON THE USE OF THE TAMPON IN THE HEMORRHAGE OF ABORTION.

BY RUFUS W. GRISWOLD, M. D., ROCKY HILL, CONN.

The authorities of fifty and more years ago, in advising us as to the treatment of hemorrhage in cases of abortion, made conspicuous mention of plugging the vagina as one of the most potent and certain means of arresting the too copious and dangerous discharge of blood. Of course, other methods were also mentioned ; but it is not a purpose of this paper to consider of those other methods, but to remark somewhat on the procedure of plugging, the modes for its performance, and the materials used.

The reader who chooses to consult some of the obstetrical writers of two to three generations ago, will find that Denman mentions "lint, sponge, or any soft substance, wet with spirits of wine, or some astringent liquor," as the material for a vaginal plug in flooding; that Burns mentions tow, or a soft napkin; that Dewees recommended a soft sponge wrung out in pretty sharp vinegar, and of a size to fill the vagina; that Leishman endorses this as one of the most simple and effectual of ways and materials; that Churchill advises a silk handkerchief or tow as better; and that other writers mention soft cloths, handkerchiefs, cotton or sheep's wool, etc., for the purpose:—the object in all being to so stuff the vaginal canal as to coagulate the blood and thus prevent its passage from the uterus. This teaching, which has come down to, and been pretty generally accepted by, the profession of the present period, has of late been put under sharp review by some modern authors, and the material and methods subjected to severe criticism. Prof. Skene, of the Long Island College Hospital, says: he has abandoned vaginal plugging altogether, in the flooding of abortions, because it controls the hemorrhage only partly, and seldom completely, is troublesome both to physician and patient to use, and must be often renewed because of the danger of septicemia. There is a deal or truth in these reasons thus given. A complete control by the ordinary vaginal tampon as ordinarily applied, is not to be had; in the majority of cases it will serve the purpose of saving the patient's life; but it is certainly attended with the risk that in some cases it will so far fail as to leave the life in great risk; and as to the troublesome part of it, it is positively not always an easy matter to thoroughly fill the vaginal canal with any of the materials herein alluded to, to the degree of entirely arresting an active flooding; nor can the procedure be always gone through with without a considerable amount of suffering by the patient. But in abandoning this mode of practice, it is broadly open to question if the methods advocated by the professor instead are not fully as uncertain as those he condemns,—they being first to apply an outward compress secured with a bandage, and if this fails to keep the bleeding within the bounds of safety, to then tampon the cervix; for, in the first place, while it might answer to trust to a compress over the vulva in some not very sharp cases, so much under the hand as to be easily seen at very limited periods of time, as for instance, an example in hospital, or one just around the corner, it is certain that no discreet practitioner would feel himself justified in trusting a bad job of the sort, which he could not hope to see again in several hours (as must frequently happen in practice,) to any sort of diaper tied on outside the door, where it would simply serve the purpose of absorbing the blood, in the same manner as it would absorb a discharge from the bladder, but would not stop the discharge; and in the second place, as regards tamponing the cervix, that would be hardly more secure, unless supplemented with a vaginal filling, since it may and not unfrequently does happen that an expulsive pain dilates still further the mouth of the womb, and either drives out the plug or leaves it to readily drop out, thus putting things in exactly the same condition as before. True, and as in addition to the insertion of a cervical tampon in the ordinary manner, obstetrician Barnes, of England, advises that the tampon be made by fastening cloths in a conical form around the end of a flexible rod, and, using this as the instrument, confine it in the vagina by the aid of tapes, so that it cannot be driven out. But this arrangement, while it may be comparatively easy of use, and may be kept from coming away, affords no protection at all against the further and continued dilatation of the neck of the womb, which in most cases will be likely to go forward with such rapidity that the plug that filled the entrance, when inserted, will, the next hour, not half fill it, and the flooding will go on around its entire circumference, and with very little diminution of its violence. If there could be certainty in any case that the hole filled by Mr. Barnes' probang-plug tampon would prove like one in a barrel, fixed, potent to resist further expansion, and so easy to be kept filled, the aspect of the whole matter would be changed, and the plan seem rational; but, as a matter of fact, the hole at the entrance to a uterus in the act of abortion is expanded somewhat, is easily expansible further, and in nine times out of ten is going to expand further, plug or no plug, till the contents are emptied out; besides which, the very pressure of the plug, producing thus a degree of irritation, is a provocative to expansion, and tends rather to aggravate than lessen the trouble against which the practitioner is contending. Despite the advice of so able a writer as Mr. Barnes, therefore, I think we shall do younger men good service by saying to them that after they have fixed a very nice cone-shaped tampon on the end of a suitable staff, and got every thing all ready to use it, they will compass a large amount of good by raising the nearest window and throwing the thing in the street. There is not the first element of safety in the arrangement Mr. Barnes proposes.

Differing from Skene and Barnes, Prof. Lusk of Bellevue, advises the vaginal tampon, countenancing the materials mentioned by earlier writers, but discarding sponge, as being the least reliable, because of its porosity. But he regards any of the materials we have named, if used after the ordinary fashion, as only temporary makeshifts, and not to be trusted if the patient is to be left for a few hours out of professional oversight. He thinks the "highest degree of safety can only be secured by the closest observance of the rules of art;" and the closest observance of the rules of art in this condition exists in packing disks of carbolized cotton behind and around the os, by the aid of a Syms speculum and other gynæcological traps, and continuing down with them till we reach the narrow portion of the vagina above the vestibule. The professor thus discards the tampons of his predecessors in midwifery as unreliable, except as temporary expedients, not to be trusted long out of observation : and unless a great deal of care be taken in the ordinary way of packing, his view is in a measure correct ; and great care in the ordinary way, as has been observed, is difficult to the attendant and troublesome to the patient.

The mode of procedure advised by Prof. Lusk, and the material indicated, commend themselves to our artistic taste and to our ideas of safety ; but the employment of them supposes first that material and tools are either at hand or can be readily had ; it supposes also proper and competent assistance, proper disposal of the patient as to light and position ; and it supposes further plenty, of time for the operation. If one could go to his case with a previous knowledge of the condition of things, and take all his traps along with him, in the same easy and collected manner as he goes to a case of ruptured perineum or of ovariotomy, the procedure recommended would be worth acceptance ; but the truth is that the vast majority of cases that come to the great body of everyday practitioners don't fall in the way. The treatment of flooding from abortion is generally an emergent treatment. You are called out to go a mile, two, five, ten miles, to see a woman supposed to be near death, frequently having no intimation even as to what may be the matter. You don't take along any Syms speculum, nor any little paper bag of carbolized cotton wadding. You find a woman in the condition of dangerous collapse from the loss of blood, and sometimes with an even chance that if she loses another teacupful before you arrest the flow, she will go off entirely. The call is for *immediate* treatment ; and the practitioner who wastes time in preparing carbolized cotton disks, syringing out the vagina, getting his patient in position for the introduction of a speculum, and all that sort of a thing, while meanwhile the woman is getting worse and worse, may not be lacking scientific attainment in his profession, but he does lack—what is of vastly more importance for the moment—common sense and the proper talent for the successful grasp of an emergency. It is the condition of emergency that requires first attention ; and if the treatment of the emergency is successful, and the hemorrhage is arrested, it is of doubtful expediency to disturb that for the purpose of putting in a better filling the next hour; if it fails, then the artistic procedure of Prof. Lusk commends itself to our attention.

After this criticism I venture an expose of my own mode of treating the flooding of abortion. When I began on these cases, thirty years ago, I followed the books, using sponge, handkerchief, cloth, etc., sometimes dry and at other times wet in some styptic wash ; but found them oftentimes difficult of introduction and not always satisfactory. I continue to use these things, when I don't have what is better. The better thing is a *junk of alum* ; the alum egg is a part of the contents of my medicine box. It is of the size of large hen-egg, oval in shape, without sharp points, but generally a little ragged. A bit of strong but not large twine is tied in a groove cut around the middle of the egg, leaving the ends so they will hang out of the vagina. To use this no preparation is necessary, no removal of the patient required. Separating the labia with the first two fingers of one hand, with the other hand the egg is introduced endways, turned half round so as to bring the long diameter across the vagina, and pushed downward and then upward against the os. If the canal is large and open, the egg can be backed with sufficient packing to hold it in position ; but in the majority of cases the supplementary support will not be needed.

This method of treatment is easy of use, speedy in operation, and effectual against further hemorrhage. The blood escaping from the uterus is very soon clotted, and an effectual tampon extending well into the os quickly formed. The size of the tampon is increased by the same process of clotting of any further blood coming against it. The vagina is also filled by the same process. This treatment has never failed me; and after its use I leave the patient with the feeling that she is safe for the next twelve or fifteen hours, so far as any danger from further bleeding is concerned. No unfavorable effects have followed the use of the alum in this way in any of the scores of cases in which it has been employed; no fevers, no septicemias, no deaths,no anything untoward; and I have never had occasion to use it the second time in any one case. The alum is a better antiseptic than any amount of carbolic acid that can be used; and for this and its powerful astringent quality, has an excellent after effect upon both uterus and vagina. Another thing in its favor, as thus employed, over any system of vaginal packing with cloths or cotton is, that micturiton is not interfered with, as it sometimes is by stuffing the vaginal canal and thus making pressure on the urethra. The alum can be removed when desired either by traction on the cord or by the introduction of the fingers; the coagulated blood fished out, the vagina syringed, and the case further treated as circumstances may require.

The use of alum is barely alluded to in some of the older writings on the flooding of the uterus; but its employment in the way I have indicated is not common, nor much known. With an impression, the outcome of considerable experience with it, that it is a valuable remedy, this advocacy is respectfully submitted.

